# Thrombocytopenia Secondary to COVID-19 Vaccination: Side Effect or Coincidence?

**DOI:** 10.7759/cureus.38219

**Published:** 2023-04-27

**Authors:** Simin Khan, Muhammed Noman Rehmani, Ahmad Kasabali, Alfred Thomas, Vinh Nguyen

**Affiliations:** 1 Internal Medicine, Louisiana State University Health Shreveport, Shreveport, USA

**Keywords:** booster vaccine, sjogren’s, immune thrombocytopenia, vaccine, covid-19

## Abstract

While widespread coronavirus disease 2019 (COVID-19) vaccination has helped achieve some control of the pandemic, vaccines have presented with side effects of their own, both common and rare. We present an unusual case of a 66-year-old who presented with severe thrombocytopenia following vaccination with the Pfizer-BioNTech mRNA vaccine.

Our patient is a 66-year-old African American female with a known history of Sjogren’s syndrome and hepatitis C who presented to our facility as a direct admit from our affiliated infusion clinic where routine lab work revealed a platelet count of 14,000. On arrival, she reported a one-month history of progressive tiredness, intermittent epistaxis, and bruising on her legs. Her physical exam was notable for multiple petechiae and non-palpable purpura on all four extremities. Further questioning revealed that she had received her COVID-19 vaccine booster (Pfizer-BioNTech) three weeks prior to presentation and that is when all the symptoms had started. Rheumatology was consulted and the patient was started on intravenous immunoglobulin infusion for two days and pulse dose prednisone. Her platelet count showed improvement after treatment, and she was discharged home with a platelet count of 42,000.

Though largely safe and efficacious, COVID-19 vaccines can present with rare systemic side effects and physicians must have a high index of suspicion and report these cases so that more data is available for interpretation.

## Introduction

During public health emergencies, if the Food and Drug Administration (FDA) can determine the health benefits of a product outweigh the known potential risks, they are able to provide an Emergency Use Authorization (EUA) to facilitate the availability of disease-preventing countermeasures. Since December 11, 2020, the Pfizer-BioNTech mRNA coronavirus disease 2019 (COVID-19) vaccine has been under EUA for those 16 and older for a two-dose primary series. On May 10, 2021, this was expanded to include those between the ages of 12 and 15 [1]. On August 23, 2021, the Pfizer-BioNTech COVID-19 vaccine was fully FDA-approved for individuals 16 years of age and older as a two-dose primary series [2]. During clinical trials, vaccine side effects were monitored by passive and active safety surveillance systems by the Center for Biologics Evaluation and Research (CBER) and the FDA [3]. One of the side effects monitored was thrombocytopenia. Immune thrombocytopenic purpura (ITP) is a hematologic disorder that affects people of all ages and genders with platelet counts <100 X 109/L and can be divided into primary and secondary manifestations. Primary is defined as isolated thrombocytopenia while secondary is due to any form of immune thrombocytopenia [4]. We report the case of a 66-year-old who presented with severe thrombocytopenia following vaccination with the Pfizer-BioNtech mRNA vaccine.

## Case presentation

We present the case of a 66-year-old female with Sjogren syndrome who presented to our infusion clinic for a scheduled rituximab treatment. Her past medical history was significant for hypertension, retroperitoneal fibrosis, and hepatitis C which had been treated. On arrival, she reported a history of progressive fatigue for the past three weeks. She also complained of intermittent epistaxis and a rash on her legs which she first noticed two days ago. Her physical exam was notable for a petechial rash on her bilateral lower extremities but no other systemic findings. Initial lab work was obtained which revealed a platelet count of 14,000 and a creatinine of 2.1. She was therefore admitted to our facility for thrombocytopenia and acute kidney injury (AKI).

On arrival to the in-patient ward, her vitals were stable and she appeared stable on the exam. Additional history obtained on arrival revealed that her home medications included hydroxychloroquine 200 mg twice daily. At this point, her thrombocytopenia was attributed to hydroxychloroquine. However, further questioning revealed that she had received her COVID-19 vaccination booster three weeks prior to this hospital admission. She had received the BNT 162b2 mRNA vaccine and had tolerated it without any issues. 

Immature platelet fraction (IPF) was elevated at 20.6. Peripheral smear was negative for schistocytes. Antinuclear antibodies (ANA) was negative. C3 was normal but C4 was elevated at 140. Her coagulation profile was normal. Vitamin B12 was elevated at 2300 and folate levels were normal. Iron and total iron-binding capacity (TIBC) levels were normal. Her flow cytometry was negative for evidence of peripheral blood involvement by acute leukemia or non-Hodgkin lymphoma. 

**Figure 1:**
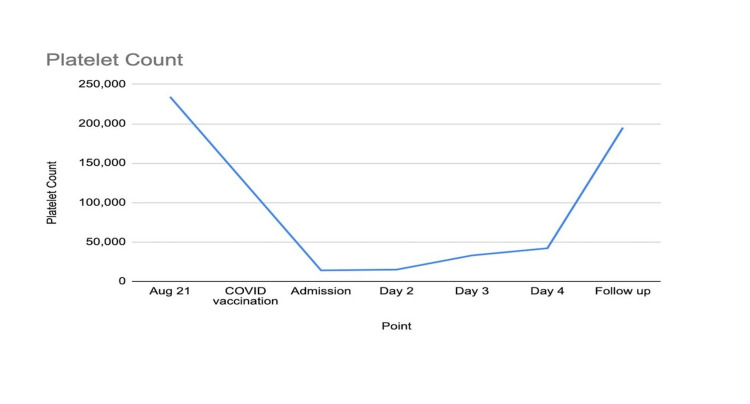
Platelet trend

Rheumatology was consulted and per their recommendations, she was initiated on intravenous (IV) high-dose steroids at 1 g daily and intravenous Immunoglobulin (IVIG) 60 g/day for two days. She was also started on IV fluids for her AKI. During her hospital stay, her platelet count was closely monitored and showed a rapid response to treatment as noted in Figure 1. As her platelet count recovered, her clinical symptoms improved too and she reported improving energy levels. 

On day three of hospitalization, her platelet count was 42,000 and she was therefore deemed stable for discharge on oral prednisolone 60 mg daily. Her hydroxychloroquine was held and she was instead placed on oral mycophenolate 500 mg twice daily. She followed up in the rheumatology clinic two weeks later. At that visit, she denied any complaints and her platelet count had improved to 195,000.

## Discussion

The severe acute respiratory syndrome coronavirus 2 (SARS-CoV-2) virus emerged as a global health crisis in December 2019 causing significant morbidity and mortality worldwide. With the rapidly evolving pathogenicity of the virus arose the need for robust vaccinations. On December 11, 2020, an EUA was issued by the FDA allowing use of the Pfizer-BioNTech COVID-19 vaccine [5]. Since then, three different kinds of vaccines have become commercially available in the United States. As of March 2022, 254,492,315 people have received at least one dose of a COVID-19 vaccine [6]. While widespread vaccination has helped achieve some control of the pandemic, vaccines have presented with side effects of their own, both common and rare.

Our patient presented with severe thrombocytopenia which was initially attributed to her underlying Sjogren’s syndrome. However, on further rheumatological evaluation, it was noted that her Sjogren’s has been largely well controlled whereas the timing of COVID-19 vaccination prior to presentation was more concerning for a vaccine side effect. Although rare, thrombocytopenia has been documented following COVID-19 vaccination.

Thrombocytopenia following vaccination is attributed to autoimmune reactions. The mechanisms postulated to induce auto-immune side effects following vaccination include the ability of some viruses to induce hyper-stimulation of the immune response and antigenic cross-reactivity secondary to molecular mimicry between human cells and specific viral components [7].

Thrombocytopenia occurs due to both humoral and cell-mediated responses leading to enhanced clearance of platelets by splenic macrophages or induction of apoptosis. One model for the pathogenesis of ITP proposed by Swinkles et al. involves an inciting event that exposes neo-epitopes on platelet antigens to immune cells. Then the loss of immune tolerance leads to the development of self-reactivity. Development of self-reactivity is more likely in individuals with existing autoimmune comorbidities indicating an existing loss of tolerance or a genetic predisposition to the development of autoimmune conditions [8]. In line with this, our patient’s preexisting Sjogren’s syndrome serves as an existing loss of tolerance, and the immune response to the vaccine serves as a possible trigger. 

## Conclusions

Vaccinations have significantly decreased the severity of COVID-19 illness, especially with the booster doses. Even though we have new COVID-19-positive cases every day, patients are largely asymptomatic or have mild symptoms. Along with creating awareness about the vaccine in the community, we should also educate them about possible side effects, so that they can seek medical help earlier. Though largely safe and efficacious, COVID-19 vaccines can present with rare systemic side effects and physicians must have a high index of suspicion and report these cases, so more data is available for interpretation.

## References

[1] (2022). FDA Approves First COVID-19 Vaccine. http://<https://www.fda.gov/news-events/press-announcements/fda-approves-first-covid-19-vaccine>.

[2] (2022). Comirnaty and Pfizer-BioNTech COVID-19 Vaccine. http://<https://www.fda.gov/emergency-preparedness-and-response/coronavirus-disease-2019-covid-19/comirnaty-and-pfizer-biontech-covid-19-vaccine>.

[3] (2022). COVID-19 Vaccine Safety Surveillance. https://www.fda.gov/vaccines-blood-biologics/safety-availability-biologics/covid-19-vaccine-safety-surveillance.

[4] Kistangari G, McCrae KR (2013). Immune thrombocytopenia. Hematol Oncol Clin North Am.

[5] (2021). Allergic reactions including anaphylaxis after receipt of the first dose of Pfizer-BioNTech COVID-19 vaccine - United States, December 14-23, 2020. MMWR Morb Mortal Wkly Rep.

[6] (2022). Stay Up to Date with COVID-19 Vaccines Including Boosters. https://www.cdc.gov/coronavirus/2019-ncov/vaccines/different-vaccines.html.

[7] David P, Dotan A, Mahroum N, Shoenfeld Y (2021). Immune thrombocytopenic purpura (ITP) triggered by COVID-19 infection and vaccination. Isr Med Assoc J.

[8] Swinkels M, Rijkers M, Voorberg J, Vidarsson G, Leebeek FW, Jansen AJ (2018). Emerging concepts in immune thrombocytopenia. Front Immunol.

